# CRKL Enhances YAP Signaling through Binding and JNK/JUN Pathway Activation in Liver Cancer

**DOI:** 10.3390/ijms25158549

**Published:** 2024-08-05

**Authors:** Marie C. Wesener, Sofia M. E. Weiler, Michaela Bissinger, Tobias F. Klessinger, Fabian Rose, Sabine Merker, Marcin Luzarowski, Thomas Ruppert, Barbara Helm, Ursula Klingmüller, Peter Schirmacher, Kai Breuhahn

**Affiliations:** 1 Institute of Pathology, University Hospital Heidelberg, 69120 Heidelberg, Germany; 2 CFMP, Core Facility for Mass Spectrometry & Proteomics at the Center for Molecular Biology (ZMBH), Heidelberg University, 69120 Heidelberg, Germanym.luzarowski@zmbh.uni-heidelberg.de (M.L.);; 3 DKFZ, German Cancer Research Center Heidelberg, 69120 Heidelberg, Germany

**Keywords:** HCC, Hippo, BioID, CRK, survival, AP1, TEAD, proteomics, MS, c-Jun

## Abstract

The Hippo pathway transducers yes-associated protein (YAP) and WW-domain containing transcription regulator 1 (WWTR1/TAZ) are key regulators of liver tumorigenesis, promoting tumor formation and progression. Although the first inhibitors are in clinical trials, targeting the relevant upstream regulators of YAP/TAZ activity could prove equally beneficial. To identify regulators of YAP/TAZ activity in hepatocarcinoma (HCC) cells, we carried out a proximity labelling approach (BioID) coupled with mass spectrometry. We verified CRK-like proto-oncogene adaptor protein (CRKL) as a new YAP-exclusive interaction partner. CRKL is highly expressed in HCC patients, and its expression is associated with YAP activity as well as poor survival prognosis. In vitro experiments demonstrated CRKL-dependent cell survival and the loss of YAP binding induced through actin disruption. Moreover, we delineated the activation of the JNK/JUN pathway by CRKL, which promoted YAP transcription. Our data illustrate that CRKL not only promoted YAP activity through its binding but also through the induction of YAP transcription by JNK/JUN activation. This emphasizes the potential use of targeting the JNK/JUN pathway to suppress YAP expression in HCC patients.

## 1. Introduction

With more than 850,000 new cases per year, liver cancer is the sixth most common type of cancer. However, since mortality nearly equals incidence, it remains one of the deadliest cancers worldwide, with its mortality ranking third among all cancer entities [1]. Although the etiology of HCC (hepatocellular carcinoma), as the most frequent primary type of liver cancer, is well understood, there is still a knowledge gap regarding the underlying molecular mechanisms causing tumor formation and progression. Recent advances in immunotherapy and systemic therapies have improved the survival of patient subgroups, but the majority of patients do not benefit [2,3]. This highlights the need for novel targeted therapies and reliable biomarkers for patient stratification.

Several studies have found that the Hippo pathway transducers YAP (yes-associated protein) and TAZ (WW domain containing transcription regulator/WWTR1) play an oncogenic role in HCC formation, progression and metastasis [4,5,6,7,8,9]. Notably, 40–60% of HCC patients show nuclear YAP enrichment, indicative of active YAP signaling [10]. Moreover, YAP and TAZ activation correlates with poor overall survival and cancer recurrence, tumor staging and dedifferentiation [11,12]. Current efforts in drug development have led to the discovery of several inhibitors targeting YAP/TAZ, which are now being tested in clinical trials and may soon become available to treat HCC patients [13,14].

Regulated by a conserved kinase cassette comprising the Hippo homologue MST1/2 (Mammalian Ste20-like kinase 1 and 2) and LATS1/2 (large tumor suppressor kinases 1 and 2), YAP and TAZ become inactivated upon phosphorylation, resulting in their cytoplasmic sequestration and eventually degradation [15]. In contrast, when the Hippo kinases are inactive, YAP and TAZ enter the nucleus, where they act as transcriptional co-activators binding to transcription factors mainly of the TEAD (*TEA domain*) family. Together with TEADs, they induce the expression of genes relevant for proliferation, cell cycle progression and cell motility [4,6,16]. Their oncogenic potential has been illustrated by several animal models with knockouts in Hippo kinases or the transgenic overexpression of constitutively active YAP mutants, which lead to tissue overgrowth and tumor formation [7,8]. Notably, the specific overexpression of mutant YAP^S127A^ in the liver not only promotes liver overgrowth, but also leads to the development of HCC nodules already after 12 weeks of transgene expression, showing that aberrant YAP activity alone sufficed to trigger tumor formation [17].

While the transcriptional target genes responsible for tumor growth are mostly known, the precise upstream regulation of YAP/TAZ is not completely understood. Originally, the central Hippo kinase cascade was postulated to be the major regulator of pathway activity. However, since it was discovered that YAP/TAZ are strong mechanotransducers responding to changes in actin cytoskeleton dynamics [18], this paradigm has been challenged, since many studies have shown actin-dependent YAP/TAZ regulation without the involvement of the canonical Hippo cascade [19,20]. It is now well accepted that YAP/TAZ activity is induced by actin polymerization and tension [21]. Animal models with knockout of the actin-capping protein CAPZB demonstrate YAP/TAZ pathway activity and the phenocopy liver overgrowth observed in YAP transgenic animals [22]. While the first evidence of the involvement of small GTPases and Src kinase has been found [23,24], it is not completely understood how information from the actin cytoskeleton is relayed to YAP/TAZ. Thus, a more detailed understanding of the complex molecular mechanisms governing YAP/TAZ activity is crucial and will help to identify targetable structures for treating patients with YAP/TAZ activation.

YAP/TAZ are usually thought to share most of their biological functions. However, knockout models of YAP and/or TAZ in the liver show that only YAP knockouts suffer from fibrosis, while the additional knockdown of TAZ even attenuates this effect [25]. Although it has been reported that YAP and TAZ share most of their transcriptional program, the exclusive functions of both proteins have also been described [6,26]. However, it is not known if YAP and TAZ have distinct interaction partners that may influence their function in HCC.

In our study, we investigated the interactomes of YAP and TAZ in HCC cells using a proximity labelling BioID approach [27] in combination with mass spectrometry. Our analysis confirmed established interaction partners like TEAD proteins and revealed previously undescribed novel candidates. We identified the adaptor protein CRKL (*CRK like proto-oncogene adaptor protein*) as a YAP-exclusive interaction partner that is overexpressed in HCC patients, correlating with a poor prognosis. CRKL inhibition affected HCC cell survival and reduced the expression of YAP/TAZ target genes. The interaction between YAP and CRKL is disrupted by actin cytoskeleton inhibitors, indicating that CRKL may activate YAP depending on the polymerization status of actin filaments. Interestingly, we also detected a JNK/JUN pathway-dependent effect of CRKL on YAP transcription, which could be a promising target to abrogate YAP overexpression in HCC patients. Taken together, we identified CRKL as a novel YAP-exclusive interaction partner that not only activates YAP but also promotes its transcription via JNK/JUN activation.

## 2. Results

### 2.1. Establishment of Biotin-Ligase Tagged YAP and TAZ

In order to identify novel binding partners of YAP and TAZ in HCC, we generated stable HLF cells expressing inducible YAP and TAZ as N-terminal fusion proteins with the biotin ligase BirA and a Flag-tag. Upon doxycycline administration, BirA-tagged YAP and TAZ were detected by Flag-tag or by the shift in protein size due to the BirA tag (Supplementary Figure S1A). The concomitant administration of biotin showed that BirA-tagged YAP and TAZ were functional and led to the biotinylation of cellular proteins, as can be seen by a “laddering” in Western Immunoblotting when biotin and doxycycline were added (Supplementary Figure S1B). Before proceeding with the actual BioID pulldown, we conducted control experiments to ensure that the BirA tag did not impair the normal biological function of YAP/TAZ. For this, we treated the cells with Cytochalasin D, an actin-depolymerizing drug known to inactivate YAP/TAZ [16]. As shown in Suppl. Figure S1C–E, Cytochalasin D led to the expected hyperphosphorylation of both endogenous but also BirA-tagged YAP/TAZ, and to their exclusion of the nucleus, confirming that BirA-tagged YAP/TAZ responded in the same manner to changes in the actin cytoskeleton as endogenous proteins. Further, we verified that BirA-tagged YAP/TAZ could interact with TEAD transcription factors by performing Co-Immunoprecipitaion (CoIP; Supplementary Figure S1F).

Together, these experiments demonstrated the functionality of the BirA-tag, as well as the normal biological behavior of BirA-tagged YAP/TAZ, which were prerequisites for the BioID pulldown assay.

### 2.2. BioID Identifies Functionally Relevant Interaction Partners of YAP/TAZ in Cancer Cells

**Figure 1:**
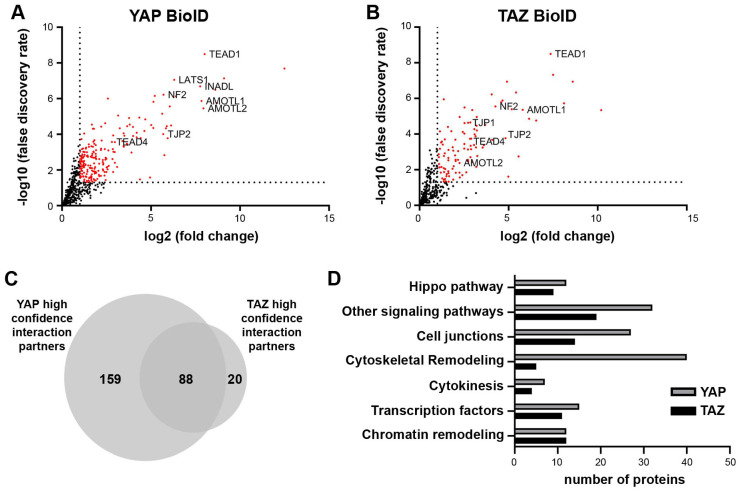
BioID identifies novel YAP/TAZ interaction partners. (**A**,**B**) Volcano plots show significant (in red) interaction partners of YAP/TAZ, as identified by the BioID pulldown. BirA-only expression was used as negative control. Well-established YAP/TAZ binding partners are labelled. (**C**) Overlap analysis of the YAP and TAZ interactome revealed high redundancy between YAP and TAZ, but also showed a vast number of exclusive YAP candidates. (**D**) Pathway enrichment analysis using the STRING database showed the involvement of YAP/TAZ interaction partners in different cancer-relevant processes.

For large-scale BioID pull-down experiments, four biological replicates of cell lines stably expressing BirA-tagged YAP/TAZ were submitted for LC/MS analysis. Only proteins significantly enriched compared to the BirA-only control (fold change ≥ 2; false discovery rate ≤ 0.05) were considered as putative YAP/TAZ interaction partners. This resulted in a YAP interactome of 247 and a TAZ interactome of 108 candidates (Figure 1A,B). Among the identified proteins were well-described interaction partners such as TEAD transcription factors, angiomotins (AMOTL1/2), LATS1 kinase and neurofibromin-2 (NF2), demonstrating the robustness of the BioID approach.

A comparison of the two interactomes showed that 81% (88/108) of the TAZ interacting candidates were also putative YAP binding partners. As the YAP interactome contained twice as many candidates, only 35% (88/247) of its binding partners were represented in the TAZ interactome (Figure 1C). This suggested that YAP may partly facilitate exclusive functions by interacting with these binding partners.

Finally, enrichment analysis using the STRING database (www.string-db.org, accessed 15 October 2020 [28]) revealed that most of the candidates were involved in cancer-relevant processes including pathways with known YAP/TAZ involvement, such as cytoskeletal remodeling, cell junctions, or chromatin remodeling (Figure 1D). Interestingly, the largest difference between YAP and TAZ binding candidates could be observed among proteins involved in cytoskeletal remodeling, suggesting that TAZ is less relevant in this process.

These results show that the applied BioID approach identified new potential YAP/TAZ interaction partners. The varying number of candidates suggests the central role of YAP in cytoskeletal remodeling.

### 2.3. CRKL Is a YAP Specific Interaction Partner

To validate the newly identified candidates, we selected potential interaction partners that were not previously described as YAP/TAZ binding proteins and that were connected to cytoskeletal remodeling or signaling. We chose VASP (Vasodilator-stimulated phosphoprotein) and RAPH1 (Ras Association and Pleckstrin Homology domains 1) as predicted YAP/TAZ interactors. In contrast, the members of the CT10 Regulator of Kinase family CRK and CRKL were chosen as YAP/TAZ and YAP-only interaction candidates, respectively.

**Figure 2:**
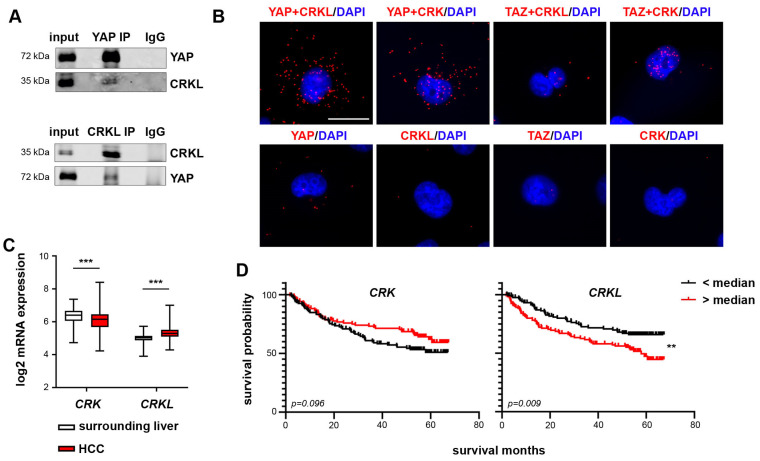
CRK and CRKL are new YAP interaction partners. (**A**) Precipitation of either endogenous YAP (upper panel) or CRKL (lower panel) and co-precipitated CRKL or YAP in HLF cells, respectively, confirming the interaction between both proteins. IgG served as negative control. (**B**) In situ PLA confirmed the predicted interactions between YAP and CRK/CRKL and TAZ and CRK in HLF cells. For TAZ and CRKL, no clear interaction was detected. Negative controls used only one primary antibody for PLA. Scale bar: 20 µm. (**C**) The transcriptome data of HCC tissue and the corresponding surrounding liver tissue (N = 242) [29] were analyzed for the mRNA expression of CRK and CRKL. While the CRK expression was decreased, CRKL expression was increased in HCC tissue. Statistical test: Mann–Whitney-U. (**D**) Patients were stratified into high- and low-expression groups using the median as a cutoff. Kaplan–Meier plots show an increased survival probability for patients with high CRK expression and a significantly decreased probability for patients with high CRKL expression. Statistical test: log-rank test. *p* ≤ 0.01 **, *p* ≤ 0.001 ***.

We verified the interactions between YAP/TAZ and VASP (Supplementary Figure S2A) and YAP and RAPH1 (Supplementary Figure S2B) by CoIP. Equally, the interaction between YAP and CRKL was also verified by CoIP (Figure 2A). Proximity ligation assays (PLAs) could also detect the interactions between YAP/TAZ and CRK (Figure 2B). However, the interaction between TAZ and CRK was weak compared to YAP and CRK. The PLA further confirmed that CRKL is a YAP-exclusive interaction partner, as no interaction was detectable for TAZ and CRKL compared to the control level.

We further analyzed the expression of the selected candidates in HCC patient data [29] and their correlation to patient prognosis. While VASP and RAPH1 expression did not significantly differ between non-tumor and tumor tissue, CRK and CRKL exhibited an opposite expression pattern. While CRK mRNA expression was significantly downregulated in HCC tissue, CRKL expression was significantly increased compared to the surrounding liver tissue (Figure 2C). Interestingly, this was reflected in the patient prognosis, as patients with high CRK mRNA levels showed an increased survival and disease-free survival probability (although not significant), while patients with high CRKL expression had a significantly lower survival probability and earlier cancer recurrence (Figure 2D, Supplementary Figure S2C). This suggested that CRK may exert tumor-suppressive functions while CRKL may act as a pro-tumorigenic factor. Due to the results from HCC patient data, we further investigated the roles of CRK and CRKL as YAP/TAZ interaction partners in liver tumorigenesis.

### 2.4. CRKL but Not CRK Influences HCC Cell Viability and Colony Formation

To study the effect of CRK and CRKL manipulation on cell survival, we selected two HCC cell lines with high protein expression (SNU182, Hep3B; Supplementary Figure S3A). Using gene-specific small interfering RNA (siRNAs), we confirmed the efficient knockdown of CRK and CRKL by real-time PCR and Western Immunoblot (Supplementary Figure S3B,C).

**Figure 3:**
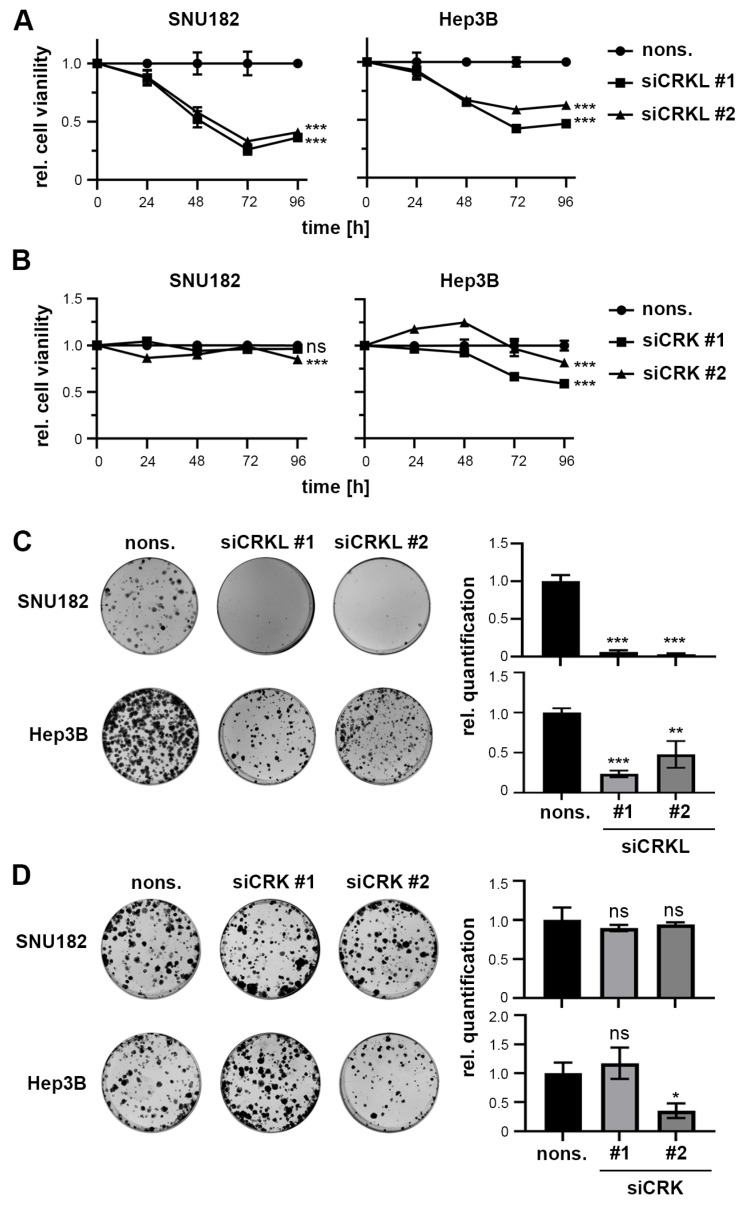
CRKL inhibition reduces cell viability. (**A**,**B**) The measurement of cell viability after different time points of CRKL and CRK inhibition in SNU182 and Hep3B cells showed reduced cell viability for CRKL inhibition. In contrast, CRK inhibition did not consistently affect cell viability. (**C**,**D**) CRKL but not CRK knockdown decreased cell survival in a colony formation assay. Exemplary images are shown. (**A**–**D**) Nonsense siRNA (nons.) transfected cells served as negative controls. (**A**–**D**) Statistical tests: Dunnett’s multiple comparison with nons. as reference. ns = not significant. *p* ≤ 0.05 *, *p* ≤ 0.01 **, *p* ≤ 0.001 ***.

The measurement of cell viability after different time points of CRK and CRKL inhibition revealed that CRKL knockdown significantly decreased cell viability by up to 70%, while CRK inhibition did not show consistent effects across cell lines and siRNAs (Figure 3A,B). This was confirmed by colony formation assays (Figure 3C,D). However, neither CRK nor CRKL affected cell apoptosis (Supplementary Figure S3D).

These results show that in vitro experiments can recapitulate the pro-tumorigenic effect of CRKL expression, while a tumor-suppressive or supporting effect of CRK could not be observed. Due to this, we decided to focus further on CRKL as the most promising YAP interaction candidate.

### 2.5. CRKL Activates YAP Signaling

CRKL is an adaptor protein that collects and transduces varieties of signals from growth factor pathways or cell adhesion molecules such as integrins to signaling pathway downstream effectors like ERK or AKT, leading to their activation [30,31]. Due to its known function as an adaptor protein, we hypothesized that CRKL might also be linked to YAP activation.

**Figure 4:**
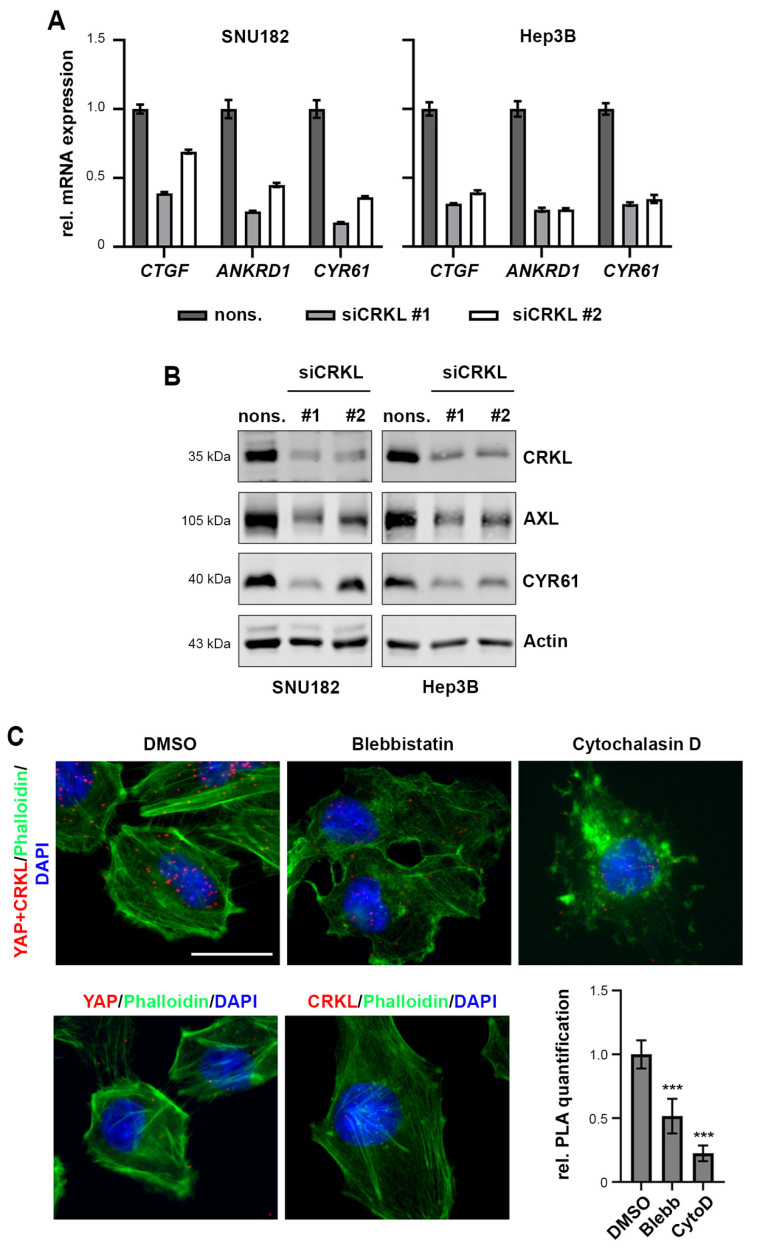
CRKL activates YAP signaling. (**A**) SiRNA-mediated inhibition of CRKL (48 h) reduced YAP target gene expression, as measured by semiquantitative real-time PCR. Gene expression was normalized to nonsense siRNA (nons.) control. (**B**) SiRNA knockdown of CRKL (48 h) reduced the expression of YAP targets AXL and CYR61, as shown by Western Immunoblot. Nonsense siRNA served as control. (**C**) Treatment with the actin inhibitors Blebbistatin (1 h) and Cytochalasin D (10 min) reduced YAP/CRKL interaction in SNU449 cells, as quantified by PLA. Phalloidin staining shows inhibition of actin cytoskeleton upon inhibitor treatments. DMSO treatment was used as control. YAP and CRKL antibody-only PLAs served as negative control for PLA. Scale bar: 20 µm. Statistical test: Dunnett’s multiple comparison with DMSO as reference. *p* ≤ 0.001 ***.

Indeed, when we inhibited CRKL by siRNAs, we detected a decrease in the mRNA and protein expression of the well-established YAP target genes CTGF, CYR61, ANKRD1, and AXL (Figure 4A,B).

To further investigate the interaction between YAP and CRKL, we used PLA after treatment with the actin polymerization inhibitor Cytochalasin D or the myosin II inhibitor Blebbistatin. Both drugs inactivate YAP by disrupting the actin cytoskeleton (Cytochalasin D) or actin-generated tension (Blebbistatin). Figure 4C shows the reduced interaction between YAP and CRKL when cells were treated with Cytochalasin D or Blebbistatin, indicating that the inactivation of YAP is accompanied by decreased CRKL binding.

From these experiments, we conclude that the actin network controls YAP/CRKL interaction and that the binding of CRKL promotes YAP activation.

### 2.6. CRKL Activates JNK/JUN Signaling to Induce YAP mRNA Expression

**Figure 5:**
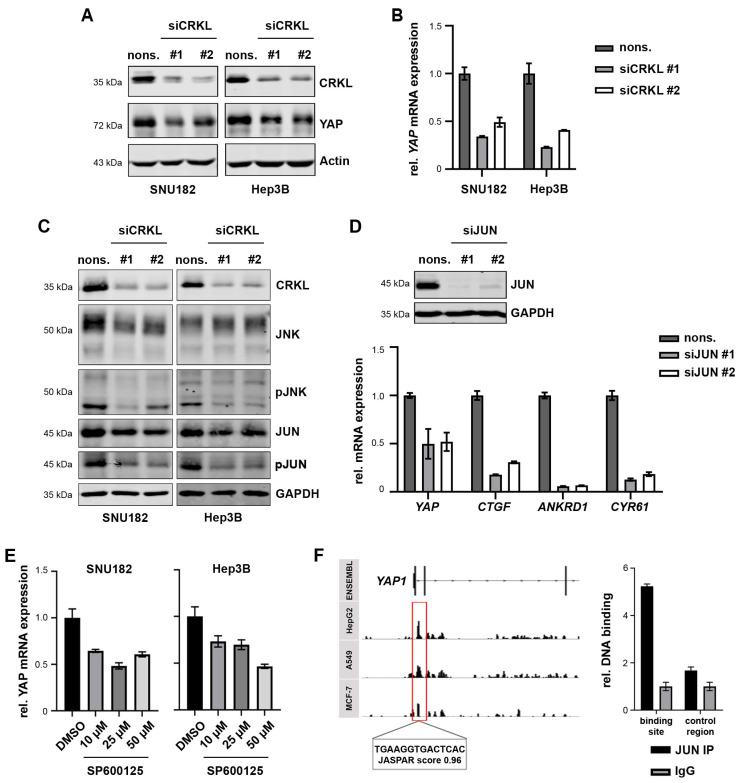
CRKL activates JNK/JUN to induce YAP transcription. (**A**) The inhibition of CRKL reduced the YAP protein level, as shown by Western Immunoblot. Actin served as the loading control. (**B**) The YAP mRNA level was decreased after the knockdown of CRKL, as measured by real-time PCR. (**C**) CRKL inhibition reduced the phosphorylated JNK (pJNK) and JUN (pJUN) levels, as shown by Western Immunoblot. GAPDH served as the loading control. (**D**) SiRNA-mediated knockdown of JUN led to a reduction in YAP mRNA, as well as a reduction in the target gene expression, as shown by real-time PCR. (**E**) Treatment with the JNK inhibitor SP600125 for 24 h reduced YAP expression, as illustrated by real-time PCR. DMSO served as control. (**F**) The scheme shows JUN ChIPseq peaks at the genomic YAP locus for three different cell lines from the ENCODE project. A predicted JUN binding site is highlighted. The bar graph shows the results from JUN ChIP with the amplification of the predicted binding site compared to IgG precipitation and a negative downstream control region. For siRNA transfection, nonsense siRNAs (nons.) served as controls.

As our data indicated, CRKL influenced YAP signaling presumably by aiding in YAP activation. However, during our inhibition experiments, we noticed that CRKL also exhibited an additional effect on YAP expression itself. When CRKL was inhibited, we detected a reduction in the YAP total protein expression (Figure 5A), as well as decreased YAP mRNA levels (Figure 5B). This led us to hypothesize that CRKL not only activated YAP signaling through binding but also through the regulation of YAP transcription.

To test this hypothesis, we analyzed the activation of different signaling pathways that may impact YAP transcription. As shown in Supplementary Figure S4, the AKT and ERK pathways were not affected by CRKL knockdown, as we did not observe changes in phosphorylated AKT or ERK levels. However, it has also been reported that the CRK family can affect c-JUN signaling via the activation of c-Jun N-terminal kinase (JNK) [32]. Indeed, CRKL inhibition strongly reduced the levels of phosphorylated JNK and JUN, while the total protein amounts were not affected (Figure 5C).

Thus, we next examined if JUN inhibition phenocopied CRKL inhibition. Upon JUN knockdown, we observed a decrease in both YAP mRNA levels and in YAP target genes (Figure 5D, Supplementary Figure S4B), which confirms that CRKL-dependent YAP transcription may be mediated by JUN. Interestingly, the inhibition of JUN had a stronger effect on YAP target genes than on YAP itself, which could be explained by several publications showing that JUN, as a part of the AP1 (*activating protein 1*) complex, cooperates in YAP-dependent target gene expression [33,34].

Using a pharmacological inhibitor of JNK, SP600125, we could further validate the connection between the JNK/JUN pathway and YAP transcription, as treatment with the inhibitor led to both reduced YAP mRNA expression and target gene expression (Figure 5E, Supplementary Figure S4C).

Subsequently, we postulated that JUN may directly induce YAP transcription. Therefore, we analyzed publicly available JUN Chromatin-Immunoprecipitation Sequencing (ChIP Seq) results from different cancer cell lines (ENCODE project [35]). As depicted in the scheme of Figure 5F, ChIP peaks were detected in all three cancer cell lines in the first intron of the YAP gene. The prediction of JUN binding sites with the JASPAR tool [36] revealed a potential JUN binding site within this region. Indeed, we amplified the binding site with a JUN ChIP, confirming that JUN directly binds the predicted binding sequence in the YAP gene.

Taken together, our data show that CRKL not only enhances YAP signaling but also induces YAP expression by activating JNK/JUN.

### 2.7. CRKL Expression Correlates with Active YAP in HCC Patients

**Figure 6:**
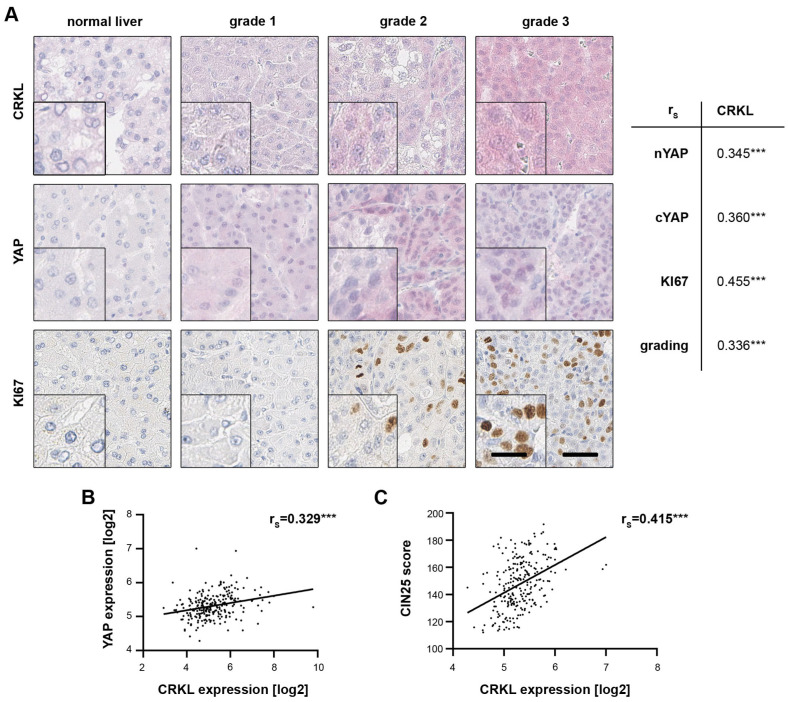
CRKL expression correlates with YAP activation in HCC patients. (**A**) The immunohistochemical staining of a tissue-microarray against CRKL, YAP and KI67 illustrated higher CRKL and YAP protein expression in HCC tissues compared to non-tumor liver tissue. The staining intensity increased with tumor grading and KI67 positivity. Three HCC samples with an increasing tumor grade, as well as a non-tumor liver sample, are shown as examples. Spearman’s rank correlation revealed significant correlations between CRKL protein expression, YAP abundance, KI67 staining and tumor grading. (**B**) Spearman’s rank correlation showed a significant correlation between CRKL mRNA expression and the YAP mRNA levels in HCC patients (N = 242) [29]. (**C**) CRKL mRNA expression correlated with a YAP target gene signature (CIN25 [4]) in HCC patients (N = 242). For Spearman’s rank correlation, a score of the target signature was calculated by summing up the normalized expression values of the 25 CIN genes. *p* ≤ 0.001 ***.

To further substantiate the connection between CRKL and YAP, we performed immunohistochemistry (IHC) on a tissue microarray (TMA) containing 40 nontumorous liver tissues, 174 cirrhotic liver tissues and 476 HCCs. By quantifying both nuclear and cytoplasmic YAP positivity, we could detect a significant positive correlation between CRKL and active, nuclear YAP. Moreover, we observed correlations with the proliferation marker KI67 and tumor grading (Figure 6A), both markers for tumor aggressiveness. This agrees with the effect of CRKL on cell viability and patient survival, as illustrated in Section 2.3 and Section 2.4.

Using transcriptome data, we correlated the expression of CRKL both with YAP mRNA levels (Figure 6B) as well as a signature (CIN25) reflecting YAP activity (Figure 6C) [4]. In both cases, a significant association was revealed.

These data confirm our in vitro analyses, showing that CRKL activates YAP signaling and correlates with tumor cell viability and aggressiveness in HCC patients.

## 3. Discussion

HCC belongs to the deadliest cancers worldwide, with its incidence still on the rise, although new treatment options are becoming available [2]. The transcriptional co-activators of the Hippo pathway, YAP and TAZ, are considered to be oncogenes that promote HCC tumor formation and progression, with up to 70% percent of patients showing nuclear positivity for either factor [26]. To date, no recurrent mutations or gene amplifications/deletions in the respective Hippo pathway genes have been found in HCC that may explain this activation. Thus, other signaling pathways as well as mechanical stimuli from cirrhotic livers may control the activity of YAP/TAZ signaling [37]. Deciphering these signals is important not only to improve our understanding of YAP/TAZ-dependent liver tumorigenesis, but also to identify novel treatment targets that affect YAP/TAZ activation apart from the canonical Hippo pathway.

In our study, we aimed to identify novel YAP/TAZ interaction partners in HCC cells that may play a role in YAP/TAZ regulation. We used proximity-labeling of YAP/TAZ interacting candidates by biotin (BioID), followed by mass spectrometric identification. This approach proved to be very robust as many well-established interaction partners were identified, such as components of the central Hippo cassette (e.g., LATS), TEAD transcription factors, as well as junctional proteins known to regulate YAP/TAZ activity (e.g., AMOTL). While most interaction partners were shared between YAP and TAZ, we identified twice as many partners for YAP than for TAZ, which was mostly involved in cytoskeletal remodeling. This suggests, in part, exclusive functions for YAP in this cellular process. Using CoIP and PLA experiments, we verified CRK and CRKL as new YAP/TAZ and YAP-exclusive interaction partners, respectively. Both proteins are adaptor proteins of the CT10 Regulator of Kinase family that share a high sequence homology and that play a role in various cellular processes, e.g., proliferation, by linking signals from different cellular receptors like receptor tyrosine kinases to downstream signaling molecules like small G-proteins [38]. Through their SH2 (Src Homology 2) and SH3 (Src Homology 3) domains, they bind to phosphorylated tyrosine residues and proline-rich sequences, respectively, the latter being present in downstream effectors like PI3-kinase or JNK [38]. Notably, YAP contains a described SH3-binding domain as well as a proline-rich motif at the N-Terminus, which is not present in TAZ [15]. These structural differences between YAP and TAZ could explain the YAP-exclusive interaction of CRKL.

Although CRK and CRKL have a high sequence overlap, different functions have been reported. While CRKL can transform fibroblasts and shows oncogenic activity, CRK fails to do so [39,40]. This is consistent with our findings in human HCC patients where CRKL is overexpressed, correlating with poor prognosis, while CRK expression is reduced, leading to a better overall survival. Moreover, our in vitro functional assays showed that CRKL but not CRK promoted HCC cell viability, which is why we have chosen to analyze CRKL further.

CRK proteins transduce signals from upstream stimuli such as integrins, via focal adhesion kinase (FAK) and Src kinase, to downstream effectors, usually leading to their activation [41]. Due to this, we hypothesized that the binding of CRKL may promote YAP activation. Using disruptors of actin polymerization and actin tension, which have extensively been shown to inactivate YAP [16,21], we analyzed the interaction between YAP and CRKL and found that YAP inactivation is accompanied by a loss of CRKL binding, suggesting CRKL may promote YAP activity since CRKL inhibition also reduced YAP target gene expression.

When activated, CRKL binds to phosphorylated tyrosine residues to transmit signals. The main substrate for those tyrosine residues is p130Cas (CRK-associated substrate)/BCAR1, which becomes highly phosphorylated by tyrosine kinases, e.g., Src and FAK, and thus provides binding sites for the SH2 domain of CRKL [42]. Interestingly, p130Cas/BCAR1 was among our YAP/TAZ candidates identified by BioID and we could verify this interaction. However, we did not follow up on p130Cas/BCAR1, since there were no correlations of BCAR1 expression with survival in HCC patients. Notably, a study in non-small cell lung cancer also identified p130Cas/BCAR1 as a YAP interaction partner, promoting YAP activation in response to FAK activation [43]. This is particularly interesting, as p130Cas/BCAR1 has been described as a tension sensor, responding to changes in the cytoskeleton during, e.g., cell spreading [44]. Combining these observations suggests that YAP is activated by cellular tension through binding to the p130Cas/BCAR1 and CRKL complex, which may act as a mechanosensor promoting YAP activity. Further studies will show if the p130Cas/BCAR1/CRKL axis is responsible for transducing mechanical signals to YAP.

Apart from activating YAP through binding, we could also show that CRKL affects YAP on a transcriptional level. By activating JNK/JUN signaling, CRKL promotes YAP transcription, inducing the expression of its own binding partner. This was also reflected by a significant correlation between CRKL and YAP expression in HCC patients. This explains how YAP levels may be elevated during tumorigenesis through upstream signals converging on CRKL. Interestingly, this may also explain the increased YAP expression levels in mouse models with elevated actin stress fibers that may induce YAP transcription through p130Cas/BCAR1-CRKL-JNK and AP1 [22]. Notably, the JNK/JUN axis provides a therapeutic target to abrogate YAP expression in HCC patients. Available inhibitors of JNK or the AP1 complex could be used to treat patients with YAP activation. Indeed, the blocking of AP1 with an inhibitor reduced YAP-dependent hepatomegaly in YAP-transgenic mice [45]. Interestingly, this study also showed that the AP1 constituent, FOS, is a transcriptional target of YAP; together with other publications, our study showed the necessity of AP1 for YAP-dependent gene regulation [33,34]. Taken together, our findings and published data highlight that inhibiting AP1 is a possible strategy to target YAP/TAZ activity in cancer patients.

## 4. Materials and Methods

Antibodies, primer and siRNA sequences can be found in the Supplementary Information in Tables S1–S5.

### 4.1. Cultivation of Cells, siRNA Transfections and Inhibitor Treatments

The HCC cell lines SNU449 and SNU182 (both ATCC) were cultured in RPMI (Sigma-Aldrich, Taufkirchen, Germany). The HCC cell line HLF (JCRB), as well as HEK293T (ATCC) cells, were cultured in DMEM (Sigma-Aldrich), while the HCC cell line Hep3B (ATCC) was cultured in MEM (Sigma-Aldrich). All cell culture media were supplemented with 10% FCS (Thermo Fisher, Darmstadt, Germany) and 1% penicillin/streptomycin (Sigma-Aldrich) and cells were maintained at 37 °C in a 5% CO_2_ atmosphere. Cells were regularly checked for mycoplasma contamination and authenticated by short tandem repeat (STR) analysis (Microsynth, Göttingen, Germany).

SiRNAs were transfected using Lipofectamine RNAiMAX (Invitrogen/Thermo Fisher transfection reagents according to the manufacturer’s instructions. Cells were seeded one day prior to transfection and harvested at the indicated time points.

Inhibitors were directly added into the cell medium at the following final concentrations: Cytochalasin D 2 µM (ENZO Life Sciences, Lörrach, Germany), Vinblastine 0.1 µM (University Hospital Pharmacy, Heidelberg, Germany), Blebbistatin 10 µM (Sigma-Aldrich), and SP600125 10–50 µM (Selleck Chemicals, Houston, TX, USA). DMSO treatment served as a control. Cells were incubated with inhibitors at 37 °C in an incubator for 1 h or 24 h in the case of SP600125.

### 4.2. Gateway Cloning

The cDNA sequences of YAP and TAZ (kind gifts of Dr Xiaolong Yang (Kingston, Canada) and Dr. Xaralabos Varelas (Toronto, ON, Canada), respectively) were cloned into pDONR plasmids, followed by LR reactions into the lentiviral pTRIPz vector, which contained N-terminal Biotin ligase BirA and a Flag tag, which were inserted into pTRIPz via the AgeI restriction site (BirA cDNA was a kind gift from Dr. Alessandro Ori, Jena, Germany). Gateway reactions were performed according to the manufacturer’s instructions (Gateway™ BP Clonase™ II Enzyme Mix and Gateway^TM^ LR Clonase^TM^ II Enzyme Mix, Thermo Fisher). Sequencing reactions were performed by Microsynth.

### 4.3. Lentiviral Transduction

For the production of lentiviral particles, HEK293T cells were seeded one day prior to transfection. For this, 10 µg of lentiviral plasmid, 8 µg of psPAX2 and 2.5 µg of PMD2.G packaging plasmids were mixed with the transfection reagent PEI (Polyehtylenimine; Polysciences, Warrington, PA, USA) in Opti-MEM (Life Technologies, Darmstadt, Germany) and distributed onto the cells. After incubation for 12–15 h in an incubator, the medium was changed. Then, 72 h after transfection, lentivirus was harvested by collecting the media and filtering it through a 0.45 µm filter to remove cells.

For the lentiviral infection of HLF cells, the medium was supplemented with polybrene (8 µg/mL, Sigma-Aldrich) and viral particles were distributed. After 24 h of incubation, the medium was exchanged and after a further 24 h of incubation, puromycin (Sigma-Aldrich) was added at a final concentration of 2 µg/mL to select stably transduced cells. To induce transgene expression, 1 µg/mL of doxycycline (Sigma-Aldrich) was added to the cells for 48 h.

### 4.4. RNA Isolation, cDNA Synthesis and Real-Time PCR

Total RNA was isolated with the EXTRACTME TOTAL RNA KIT (7Bioscience, Hartheim, Germany) according to the manufacturer’s protocol. cDNA synthesis was performed with PrimeScript RT Master Mix (TakaraBio, Saint-Germain-en-Laye, France) using 1 µg of RNA. Semi-quantitative real-time PCR reactions were set up using the Primaquant 2x qPCR-SYBR-Green-Mastermix (Steinbrenner Laborsysteme, Wiesenbach, Germany) and analyzed using the Quant Studio 3 real-time PCR system (Applied Biosystems, Darmstadt, Germany). The cycling conditions were as follows: 95 °C for 15 min, 40 cycles of 95 °C for 15 s, and 60 °C for 60 s. The product specificity was confirmed by melting curve analysis (95 °C for 15 s, 60 °C for 30 s, 60–95 °C 0.5 °C/s). Ribosomal protein L41 (RPL41) was used for normalization. Exemplary results are shown (if not otherwise stated), and these were all confirmed by independent experiments.

### 4.5. Protein Isolation, Nuclear/Cytoplasmic Fractionation and Western Immunoblotting

Cell harvest, nuclear/cytoplasmic fractionation and Western Immunoblotting has been described recently [16].

For the Phos-tag^TM^ gel preparation, 25 µM of Phos-tag^TM^ acrylamide (Wako Chemicals, Neuss, Germany) and manganese(II) chloride were added to 8% polyacrylamide resolving gel solutions, as instructed by the manufacturer.

### 4.6. Biotin Treatment and BioID Pulldown

To induce proximity-dependent biotinylation, biotin (Sigma-Aldrich) was administered to cells expressing the BirA fusion protein at a final concentration of 50 µM, followed by 24 h of incubation and harvest.

For BioID pulldown experiments, transgene expression was induced with doxycycline for 24 h, followed by addition of biotin for another 24 h. Cells were harvested in BioID lysis buffer (50 mM Tris pH 7.5, 200 mM NaCl, 0.1% SDS, 1% Triton X-100, 1 mM EDTA, 0.25% Na-Desoxycholate) and sonicated for 2x 30s, followed by centrifugation at 12,000× *g* at 4 °C for 10 min. Supernatant was applied to Pierce^TM^ Streptavidin Magnetic Beads (Thermo Fisher), which had been washed twice with BioID lysis buffer, and incubated overnight at 4 °C with rotation. Afterwards, supernatant was discarded and beads were washed twice with BioID washing buffer 1 (2% SDS), once with BioID washing buffer 2 (50 mM Tris pH 7.5, 500 mM NaCl, 1% Triton X-100, 1 mM EDTA, 6% Na-Desoxycholate), once with BioID washing buffer 3 (10 mM Tris pH 7.5, 250 mM LiCl, 0.5% Triton X-100, 1 mM EDTA, 0.5% Na-Desoxycholate) and finally once with PBS. Proteins were eluted with 25 mM biotin and Laemmli buffer. Samples were shaken for 10 min in a thermomixer and afterwards boiled at 95 °C for 5 min. Four replicates were submitted for LC/MS analysis.

### 4.7. Tryptic In-Gel Digest and LC-MS Analysis

Samples were loaded onto pre-manufactured SDS-polyacrylamide gels (Invitrogen, Waltham, MA, USA). After electrophoresis, the gels were stained with colloidal Coomassie stain (20% (*v*/*v*) methanol, 10% (*v*/*v*) phosphoric acid, 10% (*w*/*v*) ammonium sulfate, 0.12% (*w*/*v*) Coomassie G-250), cut into pieces with a scalpel, and alternately washed with 50 mM NH_4_HCO_3_ and 50 mM NH_4_HCO_3_/50% (*v*/*v*) acetonitrile. The samples were further processed using the Digest Pro MS liquid handling system. In brief, samples were reduced, alkylated and digested with trypsin. Peptides were extracted from the gel pieces with aqueous extraction buffer (2.5% formic acid, 50% acetonitrile), concentrated in a vacuum centrifuge and dissolved in 15 µL 0.1% trifluoroacetic acid.

Nano-flow LC-MS/MS was performed by coupling an Easy-nLC 1200 (Thermo Fisher Scientific, Waltham, MA, USA) to an Orbitrap Exploris mass spectrometer (Thermo Fisher Scientific). Peptide samples were dissolved in loading buffer (0.1% formic acid (FA), 2% ACN in MS-compatible H_2_O) and injected for each analysis. The samples were loaded onto an analytical column (75 µm × 30 cm, packed in-house with Reprosil-Pur 120 C18-AQ, 1.9 µm resin, Dr. Maisch) at a flow rate of 0.35 µL/min in 2% buffer A (0.1% FA in MS-compatible H_2_O). After loading, peptides were separated using a 60-min gradient from 2% to 38% of buffer B (0.1% FA, 80% ACN in MS-compatible H_2_O) at a 0.35 µL/min flow rate. The Orbitrap was operated in data-dependent mode (DDA), automatically switching between MS and MS/MS acquisition, with a *m*/*z* range of 350–1500. Full scan spectra were acquired in the Orbitrap at 60,000 resolution after accumulation to the set target value of 300% (100% = 1e6) and maximum injection time of 45 ms. Dependent mass spectra were generated for up to 20 precursors after fragmentation using higher energy collisional dissociation (HCD) at a normalized collision energy of 30% and a resolution of 15,000 after accumulation to the set target value of 100% (1e5) for a maximum of 22ms.

Raw data files were processed with MaxQuant version 1.6.12.0 for peptide identification and quantification. MS spectra were searched against the UniProt human proteome database (UP000005640_9606.fasta downloaded November 2019) and a contaminants database provided with the MaxQuant software, using the Andromeda search engine. The search parameters included: carbamidomethylation of cysteine residues as fixed modifications, acetyl (protein N-term), oxidation (M), and deamidation (Q, N) as variable modifications. Trypsin/P was used as a proteolytic enzyme, and up to 2 missed cleavages were allowed. The maximum false discovery rate for proteins and peptides was 0.01 and a minimum peptide length of seven amino acids was required. All other parameters were set to default values in MaxQuant. Quantitative normalized ratios were calculated by MaxQuant and subsequently analyzed with Perseus and STRING [28].

Sample preparation was conducted by the Proteomics Research Facility at the Center for Molecular Biology of the University of Heidelberg (ZMBH), while the LC/MS analysis was performed by the Proteomics Platform of the German Center for Lung Research (DZL) within the German Cancer Research Center (DKFZ).

### 4.8. Co-Immunoprecipitation (CoIP)

CoIP was performed as previously described [4].

### 4.9. Proximity Ligation Assay (PLA)

PLA was performed using the Duolink^®^ Proximity Ligation Assay Kit (Sigma-Aldrich). Cells seeded on coverslips were fixed with 4% paraformaldehyde for 10 min, washed four times with PBS for 5 min, followed by permeabilization for 5 min with 0,1% Triton in PBS. After washing twice with PBS for 5 min, coverslips were transferred into a wet chamber. Blocking, primary antibody incubation, probe incubation, ligation and amplification were performed according to the manufacturer’s instruction. After the final washing steps, the coverslips were mounted onto a microscopy slide using DAPI Fluoromount-G (Southern Biotech, Birmingham, UK) supplemented with Phalloidin iFluor488 (Abcam, Cambridge, UK). Fluorescence microscopy was carried out using the Olympus IX81 microscope and the Hamamatsu ORCA-R2 camera. Single antibody incubations were used as controls.

### 4.10. Cell Viability and Cytotoxicity Assays, Colony Formation Assays

The measurement of cell viability and cytotoxicity, as well as colony formation, has been recently described [16].

### 4.11. Immunohistochemistry and Tissue Microarray Analysis

The tumor samples used for the tissue microarray (TMA) analysis were resected at the University Hospital of Heidelberg. Histological classification was performed according to criteria established by experienced pathologists. The TMA contained 40 nontumorous liver tissues, 174 cirrhotic liver tissues and 476 HCCs (grading: G1 = 87, G2 = 311, G3/4 = 78). The study was approved by the institutional ethics committee of the Medical Faculty of Heidelberg University (S-206/2005).

The immunohistochemical stains for YAP and Ki67 have been described previously [4]. For CRKL, paraffin-embedded tissue sections were deparaffinized and rehydrated by the following washes: 3 × 5 min xylene, 2 × 2 min 100% ethanol, 2 min 96% ethanol, 2 min 70% ethanol, and rinsing with aqua dest. Antigen retrieval was performed in a steamer for 30 min, using Target Retrieval Solution Citrate pH 6 (Dako/Agilent, Frankfurt, Germany). Slides were then blocked with avidin for 10 min and biotin for another 10 min with TBST washing steps in between using the Avidin/Biotin Blocking Kit (Vector, Newark, NJ, USA). Samples were transferred into a wet chamber and incubated with the primary antibody at 4 °C overnight. After washing twice with TBST for 5 min, the samples were incubated with Enhancer Detection Line for 20 min. After washing twice with TBST, the samples were incubated with AP polymer Detection Line for another 20 min followed by 2 × 5 min washing steps with TBST. The samples were then developed in Permanent AP Red (AP Polymer Detection kit; DCS, Hamburg, Germany).

For the correlation of the protein expression, a score was derived for each tissue core from the following scoring system: quantity (0 = no expression, 1 = less than 1% positive cells, 2 = 1–9% positive cells, 3 = 10–50% positive cells, and 4 = more than 50% positive cells) and intensity (0 = negative, 1 = low, 2 = medium, 3 = strong). The product of those two parameters was calculated (range: 0–12). Nuclear and cytoplasmic staining scores were determined for YAP. The analysis was performed by two experienced investigators.

### 4.12. Chromatin Immunoprecipitation (ChIP)

The ChIP experiments were performed as previously described [4]. The primers for the AP1 binding sites on the YAP promoter were generated based on predictive binding sites identified by the JASPAR database [36] and compared with publicly available ChIP-Sequencing datasets [35]. As a negative control, a primer pair for a random sequence downstream of the promoter region without a potential binding site nearby was used.

### 4.13. Statistical, Correlation, Expression and Survival Data Analysis

All experiments were confirmed by two to three independent biological replicates. Data are represented as mean −/+ standard deviation. The non-parametric Mann–Whitney U test was used for the statistical comparison of two groups, while Spearman’s rank correlation coefficient (r_s_) was used for correlation analysis. All statistical tests were performed with PRISM 9 software version 9.4.1 (GraphPad). The significance levels are as follows: * *p* < 0.05, ** *p* < 0.01, *** *p* < 0.001.

Expression data from 247 HCC patients were accessed through Gene Expression Omnibus using the accession number GSE14520 (http://www.ncbi.nlm.nih.gov/geo, 15 October 2020) [29]. For survival analysis, patients were stratified into two groups (high and low) using the median as cutoff. Differences in patient survival were assessed using the log-rank test and presented as Kaplan–Meier curves.

## Data Availability

The paper and the Supplementary Materials contain all necessary information to assess the conclusions. Requests for resources and reagents will be fulfilled by the corresponding authors.

## Supplementary Materials

The following supporting information can be downloaded at: https://www.mdpi.com/article/10.3390/ijms25158549/s1.
