# Supplementary material for: CRKL Enhances YAP Signaling through Binding and JNK/JUN Pathway Activation in Liver Cancer
Source: Int J Mol Sci. 2024 Aug 5;25(15):8549. doi: 10.3390/ijms25158549 (PMC11312940; doi:10.3390/ijms25158549)
Supplement: Supplementary file 1 [file ijms-25-08549-s001.zip › ijms-3105019-supplementary.pdf]

Supplementary Information

Figure S1. BirA-tagged YAP and TAZ retain their biological function.
Figure S2. Verification of BioID candidates via co-immunoprecipitation.
Figure S3. CRKL and CRK expression in different cell lines, inhibition controls and apoptosis assay.
Figure S4. CRKL induces YAP transcription via JNK/JUN.
Table S1. Primer sequences for real-time PCR.
Table S2. siRNAs.
Table S3. List of primary antibodies.
Table S4. Secondary antibodies for WB.
Table S5. Primer sequences for ChIP real-time PCR.

Supplementary Figure S1

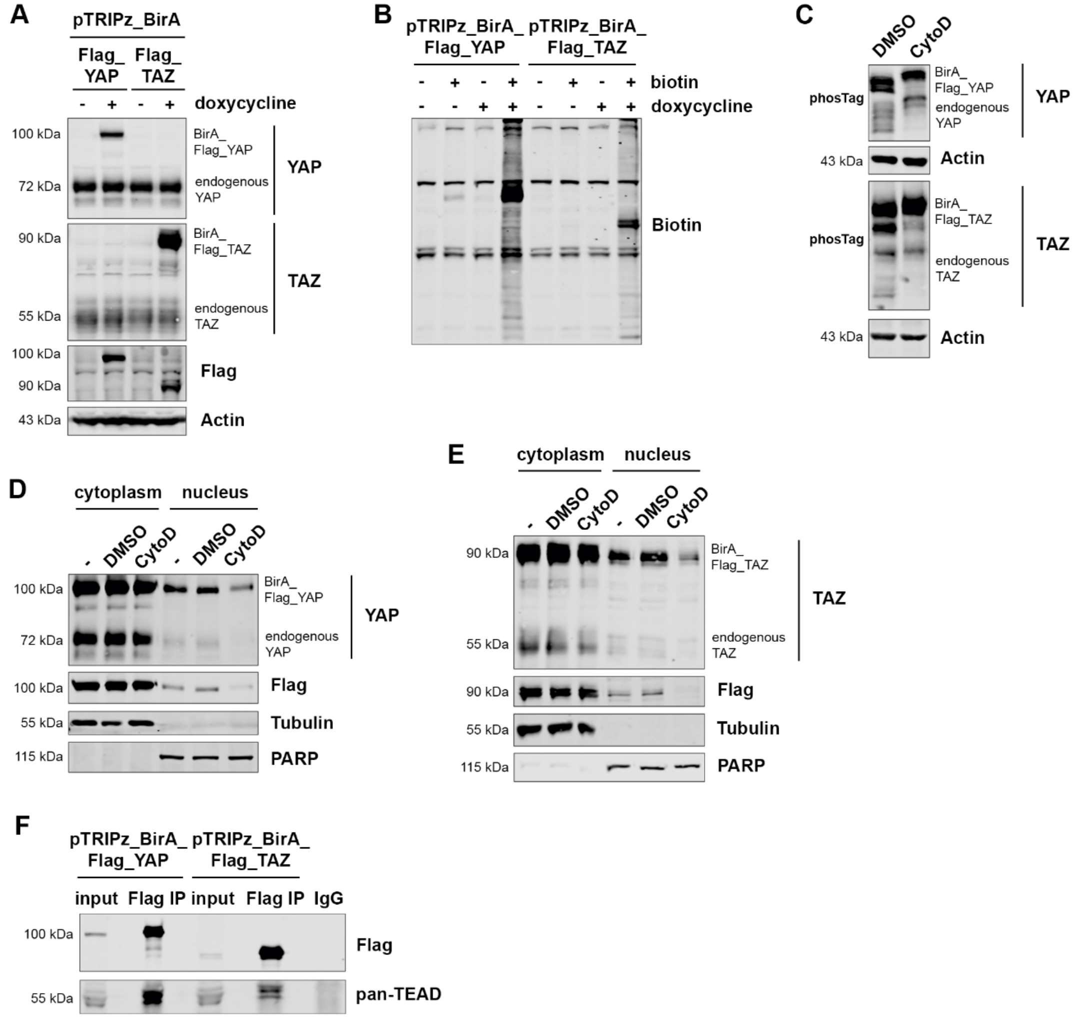

Suppl. Figure S1: **Bir-A tagged YAP and TAZ retain their biological function.**

(a) HLF cells stably expressing BirA-tagged YAP and TAZ were treated with doxycycline for 48 hours to induce protein expression. Western immunoblot shows the expression of BirA-tagged YAP/TAZ either by detecting Flag-tag or YAP/TAZ themselves, which also detects endogenous protein expression. Actin served as loading control.

(b) Only the concomitant administration of biotin (24 hours) and doxycycline (48 hours) led to the biotinylation of cellular proteins as shown by "laddering" of proteins.

(c) Treatment of doxycycline-induced BirA-tagged YAP/TAZ with Cytochalasin D (CytoD) for 1 hour led to hyperphosphorylation as detected by phos-Tag Western Immunoblot. Actin served as loading control.

(d)/(e) Nuclear/cytoplasmic fractionation after 1 hour of Cytochalasin D treatment revealed reduced nuclear levels of BirA-tagged YAP/TAZ. PARP and Tubulin served as controls for efficient fractionation.

(f) Co-Immunoprecipitation (CoIP) using Flag-tag to pulldown BirA-tagged YAP/TAZ showed co-precipitation of TEAD transcription factors. IgG served as negative control.

For (c)-(e), DMSO and untreated (-) served as negative controls.

Supplementary Figure S2

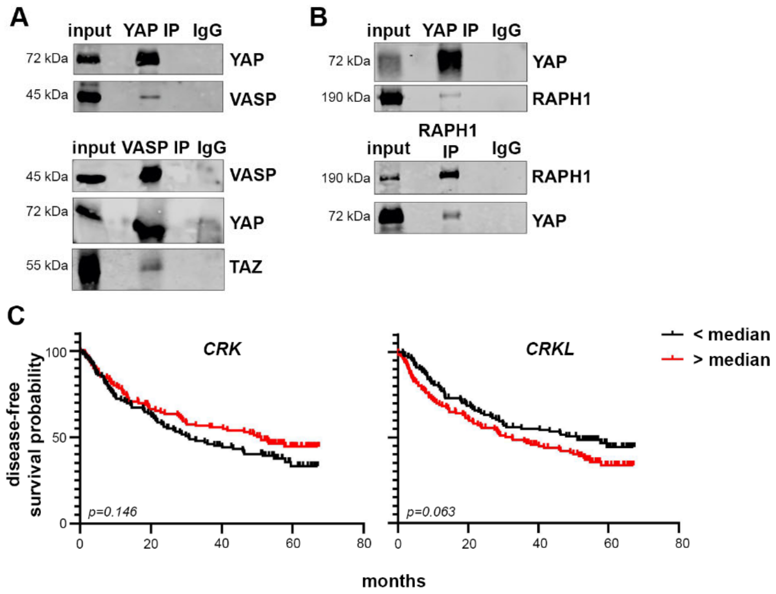

Suppl. Figure S2: Verification of BioID candidates via co-immunoprecipitation.
(a) Precipitation of either endogenous YAP (upper panel) or VASP (lower panel) co-precipitated VASP or YAP/TAZ in HLF cells, confirming the interaction between the proteins.
(b) Precipitation of either endogenous YAP (upper panel) or RAPH1 (lower panel) co-precipitated RAPH1 or YAP in HLF cells.
(c) Kaplan-Meier plots show increased recurrence-free survival probability for patients with high CRK expression and decreased probability for patients with high CRKL expression. Statistical test: log-rank test.

Supplementary Figure S3

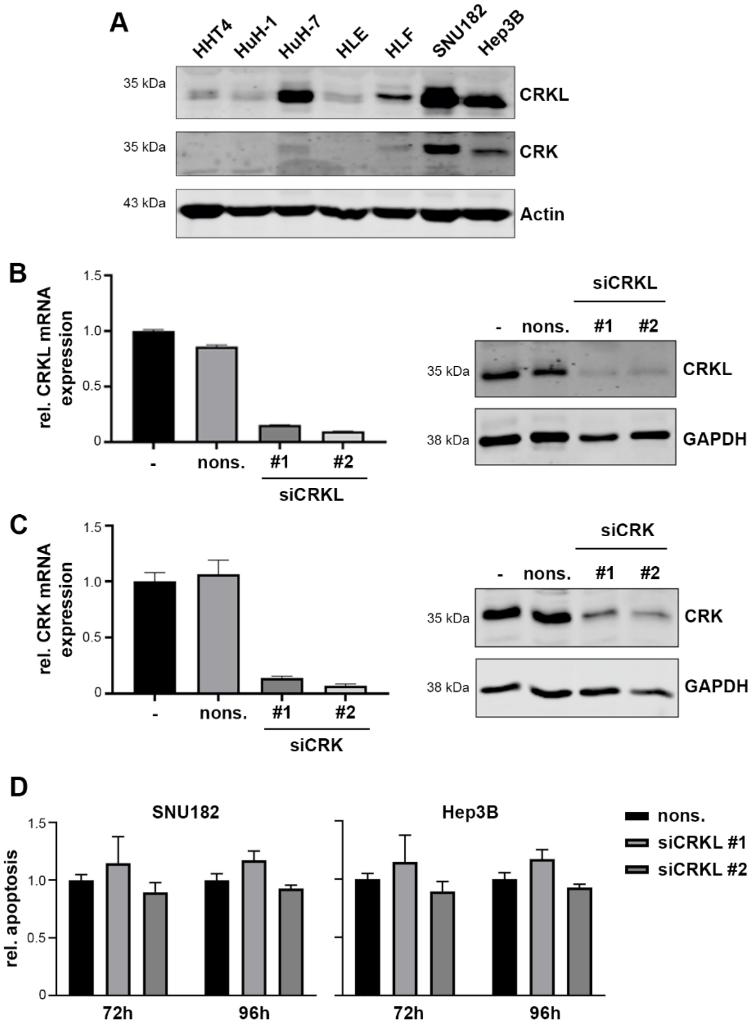

Suppl. Figure S3: CRKL and CRK expression in different cell lines, inhibition controls and apoptosis assay.

(a) Western Immunoblot showing the expression of CRKL and CRK in different HCC cell lines. Actin served as loading control.

(b)/(c) Inhibition efficiency of siRNAs targeting CRKL or CRK were confirmed by real-time PCR and Western Immunoblot. GAPDH served as loading control.

(d) Measurement of apoptosis after CRKL and CRK knockdown after 72 and 96 hours of inhibition.

Supplementary Figure S4

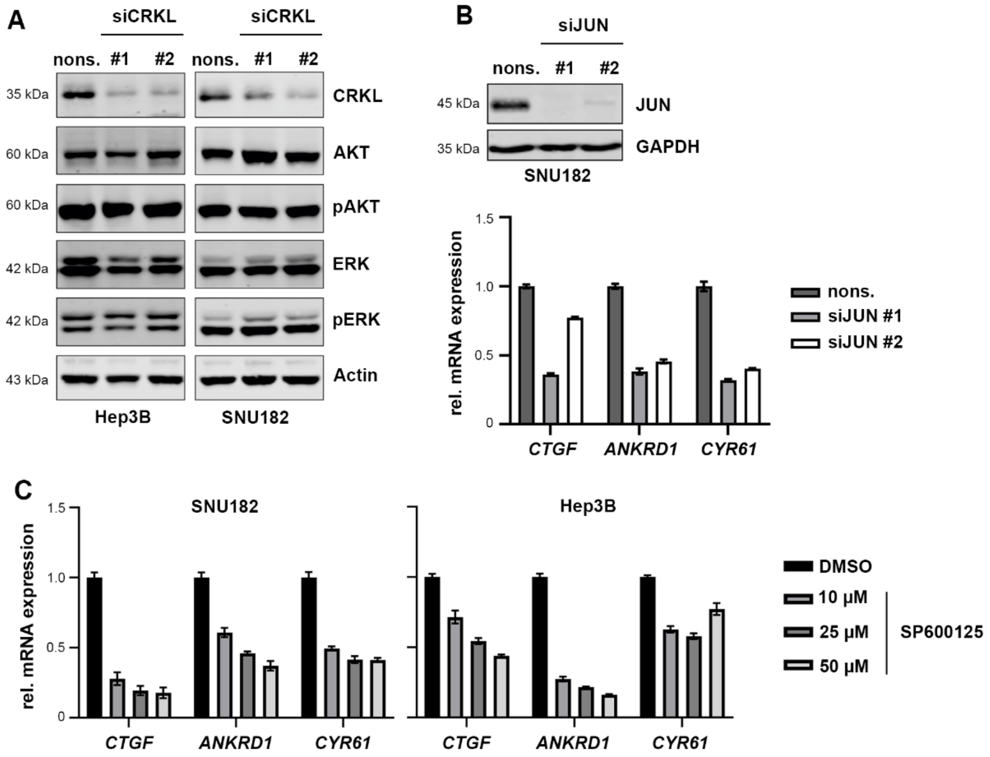

Suppl. Figure S4: CRKL induces YAP transcription via JNK/JUN..

(a) siRNA-mediated inhibition of CRKL did not decrease phosphorylated AKT (pAKT) or ERK (pERK) levels as shown by Western Immunoblot. Nonsense siRNA (nons.) served as control for transfection. Actin served as loading control.

(b) JUN inhibition reduced YAP target gene expression in SNU182 cells as measured by real-time PCR.

(c) Treatment with the JNK inhibitor SP600125 reduced YAP target gene expression as measured by real-time PCR.

Supplementary Table S1: Primer sequences for real-time PCR.

Gene	mRNA accession number	Sequence (5′-3′)
ANKRD1	NM_014391	For: AGTAGAGGAACTGGTCACTGG<br>Rev: TGGGCTAGAAGTGTCTTCAGAT
CRK	NM_005206	For: GCTCTGATTGGAGGTCGGTG<br>Rev: CAGCTGAAGTCCTCATCGGG
CRKL	NM_005207	For: GCCTTGGCATTAGAGGTTGGT<br>Rev: CGTGCGTAAAGGGGAAAAGC
CTGF	NM_001901	For: CCAAGGACCAAACCGTGG<br>Rev: CTGCAGGAGGCGTTGTCAT
CYR61	NM_001554	For: AGCCTCGCATCCTATACAACC<br>Rev: TTCTTTCACAAGGCGGCACTC
RPL41	NM_001035267	For: AAACCTCTGCGCCATGAGAG<br>Rev: AGCGTCTGGCATTCCATGTT
WWTR1/TAZ	NM_0154724	For: CAGAGAATCCAGATGGAGAG<br>Rev: GTTGACAGCAGCCTGAACTG
YAP1	NM_006106	For: CCTGCGTAGCCAGTTACCAA<br>Rev: CCATCTCATCCACACTGTTC

Supplementary Table S2: siRNAs.

siRNA	mRNA accession number	Sequence (5′-3′)
CRK #1	NM_005206	CTGCCATCTATACAATTTT
CRK #2		GACCAATCACCTCTGATAT
CRKL #1	NM_005207	CACATAGCAGACTAGAATT
CRKL #2		GCTTGACACCTTTGAATTA
JUN #1	NM_002228	GGATCAAGGCGGAGAGGAA
JUN #2		UUACUUGUCGACUCGCGCGCG
nonsense	-	UGGUUUACAUGUCGACUAA-dT-dT

Supplementary Table S3: List of primary antibodies with corresponding applications and dilutions.

Antigen	Species	Application	Dilution	Blocking solution	Company
β-Actin	mouse	WB	1:10 000	BSA/milk	MP Biomedicals
AKT	rabbit	WB	1:1000	BSA	Cell Signaling Technology #9272
Phospho-AKT	rabbit	WB	1:1000	BSA	Cell Signaling Technology #4060
Biotin	mouse	WB	1:500	BSA/milk	DAKO #M743
c-JUN	rabbit	WB	1:1000	BSA	Cell Signaling Technology #9165
Phospho-c-Jun	rabbit	WB	1:500	BSA	Sigma Aldrich #SAB4300002

CEP55	rabbit	WB	1:1000	BSA	Cell Signaling Technology #81693
CNN3	rabbit	WB	1:1000	BSA	Abcam #ab151427
CRK	rabbit	WB	1:500	BSA	Thermo Fisher Scientific #PA5-27293
		PLA	1:25		
CRKL	rabbit	WB	1:1000	BSA	Cell Signaling Technology #38710
		PLA	1:100		
		IHC	1:50		
DYDDDDK (Flag) Tag (9A3)	rabbit	WB	1:500	BSA	Cell Signaling Technology #14793
ERK	rabbit	WB	1:1000		Cell Signaling Technology #9102
Phospho-ERK	rabbit	WB	1:1000		Cell Signaling Technology #9101
FLAG (M2)	mouse	WB	1:500	BSA	Sigma Aldrich
GAPDH (14C10)	rabbit	WB	1:5000	BSA/milk	Cell Signaling Technology #2118
GAPDH	chicken	WB	1:10000	BSA/milk	EMD Millipore
JNK	rabbit	WB	1:1000	BSA	Cell Signaling Technology #9258
Phospho-JNK	rabbit	WB	1:400	BSA	Cell Signaling Technology #9251
JUN	mouse	ChIP			BD Biosciences #610326/610327
Ki67	rabbit	IHC	1:500		Abcam #15580
PARP (46D11)	rabbit	WB	1:100	BSA	Cell Signaling Technology #9532
RAPH1	rabbit	WB	1:100	BSA	Cell Signaling Technology #91138
Pan-TEAD (D3F7L)	rabbit	WB	1:1000	milk	Cell Signaling #13295
TAZ (V386)	rabbit	WB	1:1000	BSA	Cell Signaling Technology #4883
Phospho-TAZ (Ser89) (E1X9C)	rabbit	WB	1:1000	BSA	Cell Signaling Technology #59971
β-Tubulin (5H1)	mouse	WB	1:1000	BSA	BD Biosciences
VASP	rabbit	WB	1:500	BSA	Cell Signaling Technology #3132
YAP (D8H1X) XP	rabbit	WB	1:1000	BSA	Cell Signaling Technology #14074
		IHC	1:200		
YAP	mouse	PLA	1:25		Santa Cruz sc-271134
Phospho-YAP (Ser127)	rabbit	WB	1:400	milk	Cell Signaling #4911

Supplementary Table S4: Secondary antibodies used for Western Blot.

Antigen	Isotype	Application	Dilution	Company
IRDye 800CW anti-chicken	Donkey IgG	WB	1:20000	LI-COR Biosciences
IRDye 800CW anti-rabbit	Donkey IgG	WB	1:20000	LI-COR Biosciences
IRDye 680LT anti-mouse	donkey IgG	WB	1:20000	LI-COR Biosciences
IRDye 680LT anti-rabbit	donkey IgG	WB	1:20000	LI-COR Biosciences
IRDye 800CW anti-mouse	donkey IgG	WB	1:20000	LI-COR Biosciences

Supplementary Table S5: Primer sequences for ChIP real-time PCR.

Gene	Sequence (5′-3′)
JUN binding site	For: CTCATAGGCCAAGTCCTGCC<br>Rev: AGCAGCCATGTTCATATCCG
Control site	For: AGGGTCCTTAGGCAAGAGCA<br>Rev: GCAAAAACCAACTCCCTGTGAT
